# Effects of Galacto-Oligosaccharide Supplementation on Cecal Microbiota, Phospholipid and Aromatic Amino Acid Metabolism in Mice

**DOI:** 10.3390/microorganisms14030652

**Published:** 2026-03-13

**Authors:** Zisong Gao, Jue Wang, Zhiheng Cheng, Ziyang Zha, Ting Xu, Ke Yang, Tiantian Zhao, Jinglun Jiang, Pengchao Zheng, Yu Pi, Shiyi Tian

**Affiliations:** 1 College of Animal Science and Technology, Jiangxi Agricultural University, Nanchang 330045, China; 2 Key Laboratory of Feed Biotechnology of Ministry of Agriculture and Rural Affairs, Institute of Feed Research, Chinese Academy of Agricultural Sciences, Beijing 100081, China

**Keywords:** aromatic amino acid, cecal microbiota, galacto-oligosaccharide, metabolomics, phospholipid

## Abstract

Galacto-oligosaccharides (GOSs) are well-recognized for their beneficial effects on intestinal health, yet their regulatory impacts on the metabolic dynamics of other intestinal metabolites remain elusive. In this study, 24 male C57BL/6 mice were assigned to three groups: control (CON), low-dose GOS (L-GOS; 500 mg/kg body weight), and high-dose GOS (H-GOS; 800 mg/kg body weight). Following a 4-week intervention, the cecal contents were analyzed to characterize the bacterial community structure and metabolic profiles. Results indicated that GOS supplementation significantly increased the ACE and Chao1 indices of cecal bacteria. Specifically, L-GOS led to notable enrichment of the *[Eubacterium] brachy* group, *Coriobacteriaceae UCG-002*, *Faecalimonas*, and the *[Eubacterium] siraeum* group, whereas H-GOS significantly increased the abundance of *Clostridium*, *Ruminiclostridium*, *Thomasclavelia*, *Adlercreutzia*, and *Faecalimonas*. Metabolomic profiling revealed that L-GOS profoundly reduced levels of phosphatidylethanolamine, phosphatidylcholine and their downstream metabolites, while inhibiting the conversion of sphingolipids to ceramides. The changes in phospholipid derivatives imply enhanced intestinal epithelial integrity, supporting intestinal homeostasis. GOS intervention also decreased phenylacetic acid content. L-GOS increased the 4-hydroxyphenylpyruvic acid content, whereas H-GOS reduced 4-hydroxyphenyllactic acid levels. Notably, H-GOS significantly up-regulated the production of indole-3-acetic acid, a tryptophan-derived microbial metabolite with multiple biological activities. Collectively, these findings provide insights and potential targets for future research on GOS application in intestinal health interventions.

## 1. Introduction

Functional oligosaccharides represent a class of crucial prebiotic components, and recent advances in synthetic technology have facilitated their extensive application in maintaining host health [1], regulating intestinal microecology [2], and serving as adjuvant therapeutics for various diseases [3]. Galacto-oligosaccharides (GOSs), naturally derived functional oligosaccharides composed of 1–5 galactose monomers with a terminal glucose residue, are linked via Galβ(1→3), Galβ(1→6), or Galβ(1→4) glycosidic bonds [4]. Resistant to digestion in the upper gastrointestinal tract, GOSs reach the hindgut and act as selective nutritional substrates for beneficial gut microbiota [4]. Due to their structural similarity to human milk oligosaccharides (HMOs) and their ability to promote colonization of *Bifidobacterium* and *Lactobacillus* in infants, GOSs are commonly incorporated into infant formula as HMO mimics [4,5]. Over the past decade, improvements in industrial production efficiency have facilitated extensive investigation into prebiotic functions of GOSs, demonstrating benefits such as promoting intestinal development [6], regulating immune homeostasis [7], enhancing calcium absorption [8], ameliorating neurodegeneration [9], and alleviating alcohol-induced liver injury [10]. Thus, as a high-quality prebiotic, GOSs have expanded in the food industry and in applications related to human health.

Numerous studies have established that the prebiotic effects of GOSs are largely attributed to their ability to stimulate the production of short-chain fatty acids (SCFAs) in the hindgut [4,11]. Intestinal epithelial cells rapidly uptake SCFAs, which lowers their luminal concentration and thereby sustains favorable fermentation kinetics [12]. However, the hindgut microbiota constitutes a complex microecosystem, and bacterial SCFA fermentation involves intricate cross-feeding interactions that generate a diverse array of metabolic by-products, which collectively shape the metabolic profile of the microbial community [13]. Additionally, GOS supplementation has been reported to alter dry matter digestibility in the terminal ileum of animal models [14], and these undigested nutrients subsequently serve as key substrates for microbial fermentation in the hindgut. These studies strongly indicate that GOS may exert regulatory effects on the composition of intestinal microbiota-derived metabolites beyond SCFAs. In recent years, research on gut microbiota-derived metabolites has trended toward comprehensiveness and precision. A growing body of evidence has illuminated the association between gut microbial fermentation products of lipid and protein nutrients and host intestinal health [15]. For instance, previous studies have shown that *Desulfovibrio*-derived phosphatidylethanolamine and phosphatidylcholine promote the activation of γδ T cells [16]. In contrast, indole-3-acetic acid, a gut microbiota-derived metabolite of aromatic amino acids, exhibits diverse biological activities, including anti-inflammatory, anti-tumor, anti-obesity, hepatoprotective, enteroprotective, and neuromodulatory effects [17]. Although previous work examined the effects of GOS on bacterial bile acid metabolism [7], its modulation of other bacterial metabolites remains poorly understood.

Therefore, we hypothesize that GOS may significantly reshape the metabolic profile of hindgut bacteria beyond SCFA production. Unlike the gas chromatography–mass spectrometry (GC-MS) platform commonly employed for SCFA quantification, the present study utilized a liquid chromatography–mass spectrometry (LC-MS)-based untargeted metabolomic analysis to characterize the cecal metabolic profile of mice supplemented with GOS via drinking water. Furthermore, to clarify whether alterations in the cecal metabolic profile are dependent on GOS intake, we investigated the effects of different GOS doses on the composition of the cecal metabolic profile, based on the safe daily intake of GOS for humans recommended by the U.S. Food and Drug Administration.

## 2. Materials and Methods

All sample collections were performed in accordance of the Institutional Animal Care and Use Committee of Jiangxi Agricultural University (No. JXAULL-20220627).

### 2.1. Animal Experimental Design and Sample Collection

A total of 24 four-week-old male C57BL/6 mice (Hunan SJA Laboratory Animal Co., Ltd., Changsha, China) were randomly assigned to three groups: control (CON), low-dose GOS (L-GOS, 3 mg/mL), and high-dose GOS (H-GOS, 5 mg/mL). Based on daily water intake, the estimated GOS consumption was approximately 500 mg/kg body weight/day for the L-GOS group and 800 mg/kg body weight/day for the H-GOS group, both within the safe dosage range recommended by the FDA. After one week of adaptation, GOS was administered via drinking water for 4 weeks. Food-grade GOS (~90% purity) was obtained from Quantum Hi-Tech Biological Co., Ltd. (Guangzhou, China), with composition detailed in Tian et al. [18]. Mice were housed under standard conditions (22–25 °C, 50% humidity, 12 h light/dark cycle) with ad libitum access to food and water. On day 28, mice were euthanized by cervical dislocation, and cecal digesta were collected and stored at −80 °C.

### 2.2. DNA Extraction, 16S rRNA Gene Amplification, and High-Throughput Sequencing

Microbial DNA was extracted using a FastPure Stool DNA Isolation Kit (MJYH, Shanghai, China). The V3-V4 region of the 16S rRNA gene was amplified with universal primers (F 5′-ACTCCTRCGGGAGGCAGCAG-3′ and R 5′-GGACTACCVGGGTATCTAAT-3′) and sequenced on an Illumina Nextseq2000 platform (Majorbio Bio-Pharm Technology Co., Ltd., Shanghai, China). DNA samples were stored at −80 °C until further 16S rRNA MiSeq sequencing. The PCR process was performed as described in a previous study [7]. PCR products were detected by 2% agarose gel electrophoresis and purified using the AxyPrep DNA Gel Extraction Kit (Axygen Biosciences, Union City, CA, USA). Purified products were pooled in equimolar amounts and paired-end sequenced on an Illumina Nextseq2000 platform (Illumina, San Diego, CA, USA) according to standard protocols by Majorbio Bio-Pharm Technology Co., Ltd. (Shanghai, China).

### 2.3. Data Processing and Bioinformatics Analysis

Raw FASTQ files followed Tian’s method. The most abundant sequence for each operational taxonomic unit (OTU) was selected as a representative sequence. Bioinformatics analysis of the cecal microbiota was carried out using the Majorbio Cloud platform v1.0 (https://cloud.majorbio.com, accessed on 23 October 2025). Diversity of cecal bacteria (indices of Shannon, Simpson, Ace and Chao) was assessed using MOTHUR v.1.30.1. Similarity among microbial communities in different samples was determined by principal coordinate analysis (PCoA) based on Bray–Curtis dissimilarity using the Vegan v2.5-3 package.

### 2.4. Sample Preparation for LC-MS Metabolomics Analysis

A total of 50 mg of cecal digesta was weighed into a 2 mL centrifuge tube, followed by the addition of one 6 mm diameter grinding bead. Metabolite extraction was performed following the protocol described by Wu et al. [19]. Briefly, extraction solution containing L-2-chlorophenylalanine was added to the tube. The sample mixture was homogenized using a cryogenic tissue grinder, then subjected to low-temperature ultrasonic extraction. Subsequently, the sample was incubated at −20 °C for 30 min, centrifuged at 13,000× *g* and for 15 min at 4 °C, and the resulting supernatant was transferred to an injection vial with an inner insert for instrumental analysis.

### 2.5. LC-MS/MS Analysis

LC-MS/MS analysis of the samples was performed on a Thermo Scientific UHPLC-QExactiveHF-X system (Shanghai Majorbio Bio-pharm Technology Co., Ltd., Shanghai, China). Three microliters of the sample was separated on an HSST3 column (100 mm × 2.1 mm i.d., 1.8 μm) and then introduced into the mass spectrometer for detection. Mobile phase A is 95% water + 5% acetonitrile (containing 0.1% formic acid), and mobile phase B consisted of 47.5% acetonitrile + 47.5% isopropanol + 5% water (containing 0.1% formic acid). The flow rate is 0.40 mL/min, and the column temperature is 40 °C. Sample mass spectrometry signal acquisition was performed in both positive and negative ion scanning modes, with a mass scanning range of 70–1050 *m*/*z*. The sheath gas flow rate is 50 psi, the auxiliary gas flow rate is 13 psi, the auxiliary gas heating temperature was 425 °C, the ion spray voltage was set to 3500 V (positive mode) or −3500 V (negative mode), the ion transmission tube temperature is 325 °C, and the normalized collision energy is 20–40–60 V in a 3-step cycle. The resolution of the first mass spectrometer scan was 60,000, and that of second scan was 7500.

### 2.6. Metabolite Identification and Analysis

After LC-MS data were collected, raw data were imported into the metabolomics processing software Progenesis QI v3.0 (Waters Corporation, Milford, CT, USA) for baseline filtering, peak identification, integration, retention time correction, and peak alignment. A data matrix of retention time, mass-to-charge ratio, and peak intensity was obtained. MS and MS/MS mass spectrometry information were matched with the HMDB (http://www.hmdb.ca/, accessed on 23 October 2025) and Metlin (https://metlin.scripps.edu/, accessed on 23 October 2025) databases, as well as Majorbio’s in-house database. The data matrix after library matching was uploaded to Majorbio’s cloud platform (cloud.majorbio.com, accessed on 23 October 2025) for analysis. Data preprocessing was performed by Ma et al. [20]. The ropls package (Version 1.6.2) in R was used to perform principal component analysis (PCA) and orthogonal partial least squares discriminant analysis (OPLS-DA) on the preprocessed data matrix, and 7-fold cross-validation was used to evaluate model stability. Differential metabolites were annotated for metabolic pathways using the KEGG database version 2025 (https://www.kegg.jp/kegg/pathway.html, accessed on 23 October 2025) to identify the pathways involved. Pathway enrichment analysis was performed using the Python v3.14.3 scipy.stats package, and Fisher’s exact test was used to identify biological pathways most relevant to the experimental treatment.

### 2.7. Statistical Analysis

Data on α-diversity were analyzed using SPSS software (SPSS version 20.0) and expressed as means ± standard deviation (SD). For the multiple comparisons of microbiota, we used the Kruskal–Wallis test and corrected the *p*-values with FDR correction by the Benjamini–Hochberg method. The PCA was conducted based on the Bray–Curtis distance to compare groups of samples, and then the analysis of molecular variance (AMOVA) was conducted based on an unweighted distance to assess significant differences among samples by using mothur. Linear discriminant analysis effect size (LEfSe) (http://huttenhower.sph.harvard.edu/LEfSe, accessed on 28 October 2025) was performed to identify significantly abundant bacterial genera among different groups (LDA score > 2, *p* < 0.05). The data of metabolomics were analyzed using an independent samples *t*-test. Statistical significance was defined as *p* < 0.05. Significantly different metabolites were selected based on the variable importance in the projection (VIP) value and the *p*-value from Student’s *t*-test obtained from the OPLS-DA model. Differential metabolites were identified using a multi-criteria threshold (*p* < 0.05, up regulation or down regulation fold change > 1, and VIP > 1).

## 3. Results

### 3.1. GOS Supplementation Increases Cecal Bacterial Richness in Mice

**Figure 1:**
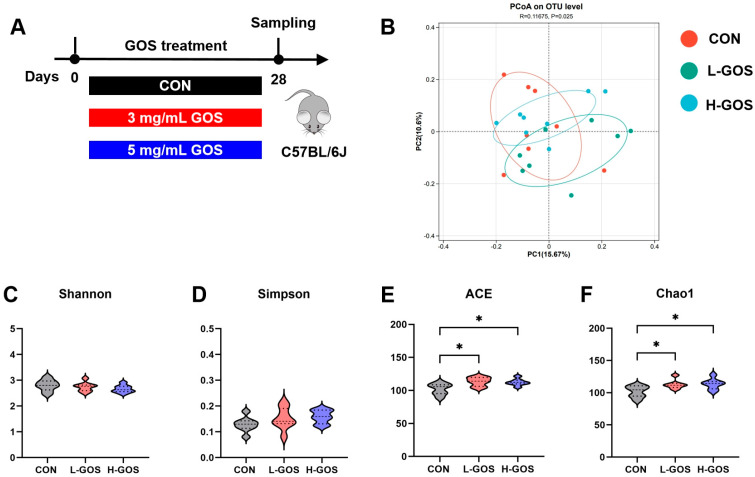
GOS supplementation altered the cecal bacterial community construction in mice (*n* = 8). (**A**) The experimental timeline. The whole experimental period was four weeks. During the experiment, mice were supplemented drinking water with different GOS concentrations. (**B**) PCoA profile of the CON, L-GOS, and H-GOS groups in cecal content. (**C**–**F**) Indices of cecal content bacterial community. All the data are expressed as Mean ± SD. * represents *p* < 0.05.

After quality control of 16S rRNA sequencing, a total of 1,488,037 high-quality sequences were obtained across all samples, with an average of 62,001 sequences per sample. The average number of OTUs per sample was 494 with a 97% sequence similarity threshold. PCoA revealed significant separation between the cecal bacterial communities of the CON and GOS-treated mice (*p* < 0.05; Figure 1B). In contrast, no significant separation was observed between the L-GOS and H-GOS groups. Changes in bacterial diversity indices within each group were also assessed (Figure 1C–F). Compared with the CON group, GOS treatment at different doses did not significantly affect the Shannon or Simpson indices (Figure 1C,D). However, it significantly increased the ACE and Chao1 indices (*p* < 0.05; Figure 1E,F).

### 3.2. GOS Supplementation Alters the Cecal Bacterial Composition in Mice

**Figure 2:**
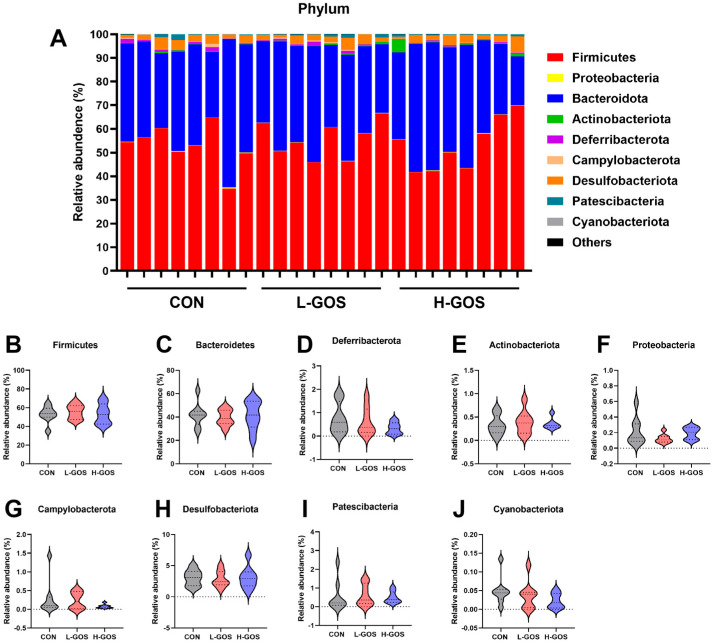
Effects of GOS supplementation on the relative abundance of bacterial phyla in cecal content (*n* = 8). (**A**) The relative abundance of dominant bacterial phyla in the cecal content of each mouse per group. (**B**–**J**) The relative abundance of Desulfobacterota, Actinobacteriota, Proteobacteria, Campylobacterota, and Cyanobacteria in cecal content. All the data are expressed as Mean ± SD.

Taxonomic classification identified nine bacterial phyla, among which Firmicutes, Bacteroidetes, and Desulfobacterota were predominant in the mouse cecal content, collectively accounting for over 90% of relative abundance (Figure 2A). Statistical analyses of these phyla revealed that neither L-GOS nor H-GOS significantly affected the relative abundances of Firmicutes, Bacteroidetes, Desulfobacterota, Actinobacteriota, Proteobacteria, Campylobacterota, or Cyanobacteria in cecal contents (*p* > 0.05; Figure 2B–J).

**Figure 3:**
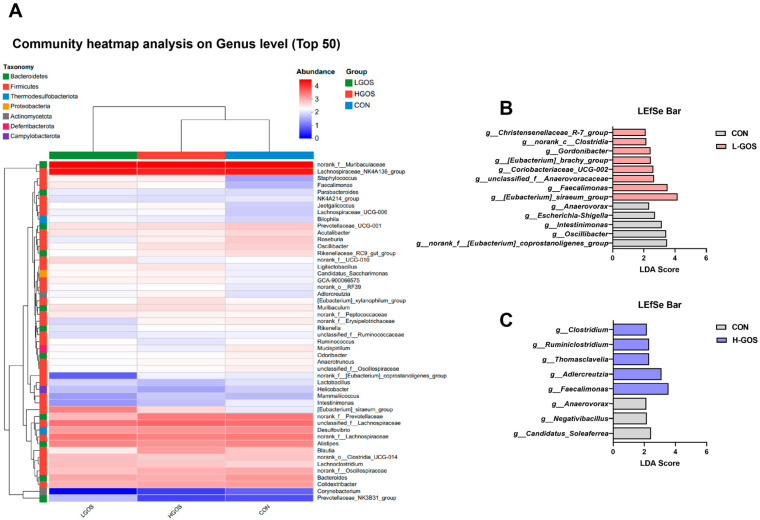
Effects of the GOS supplementation on the relative abundance of dominant bacterial genus in cecal content of mice (*n* = 8). (**A**) Heat map cluster analysis of the abundance of the cecal dominant genera (top 50 by average abundance ranking). (**B**) Marked differences in the abundance of cecal bacterial members at genus levels between CON and L-GOS groups obtained by using the linear discriminant analysis (LDA) effect size (LEfSe) method; *p* < 0.05, LDA > 2. (**C**) Marked differences in the abundance of cecal bacterial members at genus levels between CON and H-GOS groups obtained by using the LEfSe method; *p* < 0.05, LDA > 2.

Euclidean hierarchical clustering analysis of top 50 most abundant genera showed that the genus level abundance profile in L-GOS mice was markedly different from that of CON mice, whereas the H-GOS mice exhibited a profile similar to CON mice (Figure 3A). LEfSe analysis identified differentially abundant genera between L-GOS and CON groups. Genera such as *Christensenellaceae R-7* group, *norank Clostridia*, *Gordonibacter*, *[Eubacterium] brachy* group, *Coriobacteriaceae UCG-002*, *unclassified Anaerovoracaceae*, *Faecalimonas*, and *[Eubacterium] siraeum* group were significantly enriched in the L-GOS group (LDA score [log10] > 2; Figure 3B). In contrast, genera including *Anaerovorax*, *Escherichia-Shigella*, *Intestinimonas*, *Oscillibacter*, and *norank [Eubacterium]coprostanoligenes_*group were significantly enriched in the CON group (Figure 3B). Significant differences in bacterial genera were also observed between H-GOS and CON groups (Figure 3C). Genera *Clostridium*, *Ruminiclostridium*, *Thomasclavelia*, *Adlercreutzia*, and *Faecalimonas* were enriched in H-GOS mice, whereas *Anaerovorax*, *Negativibacillus*, and *Candidatus Soleaferrea* were enriched in CON mice (Figure 3C).

### 3.3. GOS Supplementation Changes Cecal Metabolic Profile in Mice

**Figure 4:**
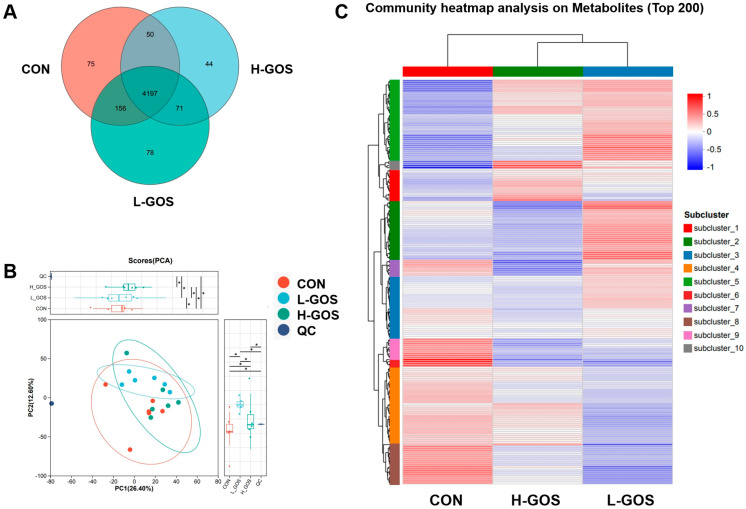
GOS supplementation altered the metabolic profile composition in the cecal content of mice (*n* = 6). (**A**) Venn of identified metabolites among the CON, L-GOS and H-GOS groups. (**B**) Principal component analysis (PCA) profiles among the CON, L-GOS and H-GOS groups. (**C**) Heat map cluster analysis of the top 200 metabolite (ranked by their abundance in each group) expression levels among the CON, L-GOS and H-GOS groups. All the data are expressed as Mean ± SD. * represents *p* < 0.05.

To balance the cost of replicates and the number of replicates necessary, we randomly selected six samples per group for metabolite analysis. Metabolites were detected in both positive (ESI+) and negative (ESI−) ionization modes, yielding 2256 and 2557 metabolites, respectively, across the CON, L-GOS, and H-GOS groups (Supplementary Tables S1 and S2). Venn diagram analysis indicated that 4197 metabolites were shared among all groups, while 75, 44, and 78 metabolites were unique to the CON, L-GOS, and H-GOS groups, respectively (Figure 4A). Cluster analysis of the top 200 metabolites grouped them into 10 subclusters based on similarities in expression patterns relative to the CON group (Figure 4B). Notably, L-GOS and H-GOS groups clustered more closely together than either did with the CON group. Although PCA score plots (PC1 = 17%, PC2 = 14.4%) did not clearly separate the GOS-treated groups from the CON group (Figure 4C), PLS-DA score plots showed clear separation between both GOS-treated groups and the CON group (Figure 4C).

**Figure 5:**
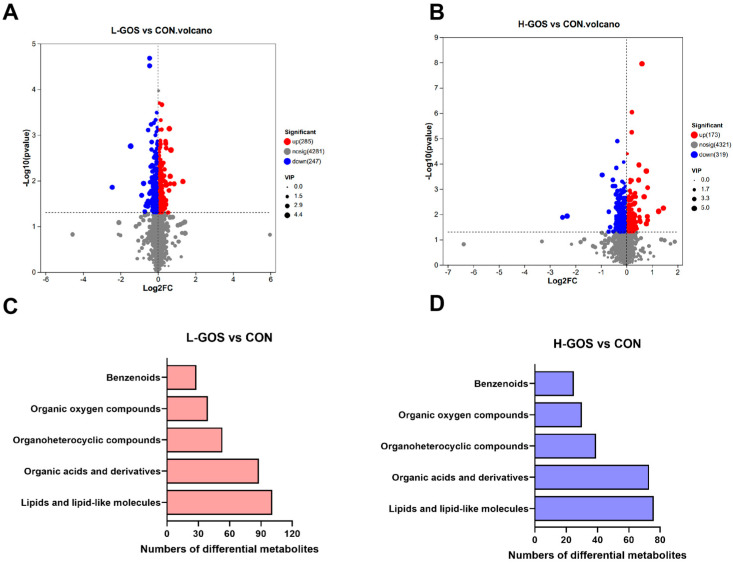
The effects of GOS supplementation on differential metabolites in cecal content of mice (*n* = 6). (**A**,**B**) Volcano plot analysis of up/down-regulated differential metabolites from L-GOS/CON and H-GOS/CON comparison groups (the red points represent proteins that have been significantly up-regulated, the blue points reflect significantly down-regulated proteins, and the gray points show no quantitative information on protein). (**C**,**D**) Classification of differential metabolites between the L-GOS/CON groups or the H-GOS/CON groups.

Volcano plot analysis revealed 532 differential metabolites between L-GOS and CON groups, with 285 up-regulated and 247 down-regulated in L-GOS mice (Figure 5A). Between H-GOS and CON groups, 492 metabolites were differentially expressed, of which 173 were up-regulated and 319 down-regulated in H-GOS mice (Figure 5B). In the global metabolomic differential analysis, 101 lipids and lipid-like molecules were identified between L-GOS and the CON groups, accounting for 19% of the total differential metabolites (Figure 5C). Meanwhile, 110 small peptides (dipeptides or tripeptides) with significantly altered abundances were identified between the H-GOS and CON groups (Figure 5D), representing the largest proportion of differential metabolites (22.3%). Detailed information of the differential metabolites, including compound names, *p*-values, VIP scores, and fold changes, is provided in Supplementary Tables S1 and S2.

### 3.4. GOS Supplementation Modulates the Cecal Content of Phospholipid Derivatives in Mice

**Figure 6:**
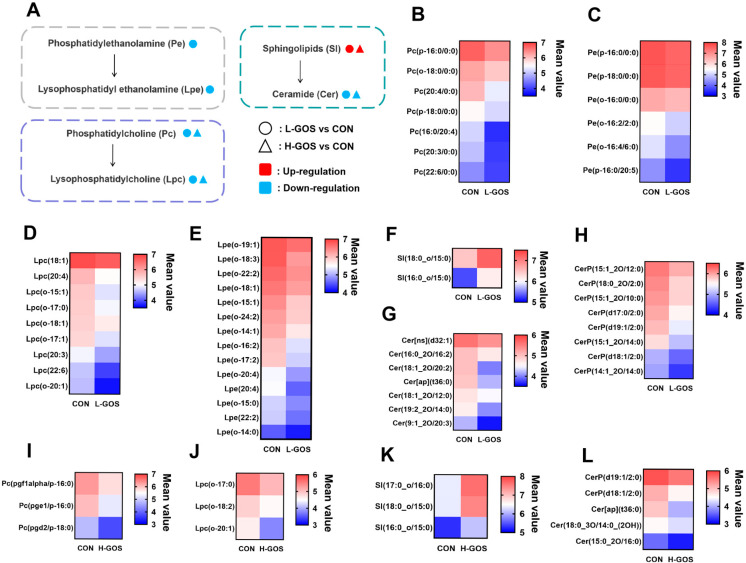
The effects of GOS supplementation on the concentrations of cecal phospholipid derivatives in the mice (*n* = 6). (**A**) Schematic overview of important metabolites and major metabolic pathways associated with phospholipid derivative metabolism. Circle represents L-GOS vs. CON; triangle represents H-GOS vs. CON; red represents up-regulated; blue represents down-regulated. (**B**,**C**) Changes in the phosphatidylethanolamine (Pe) and phosphatidylcholine (PC) between the L-GOS and CON groups. (**D**,**E**) Changes in the lysophosphatidylcholine (Lpc) and lysophosphatidylethanolamine (Lpe) between the L-GOS and CON groups. (**F**–**H**) Changes in the sphingolipids (Sl), ceramide (Cer) and ceramide phosphate (CerP) between the L-GOS and CON groups. (**I**,**J**) Changes in the Pc and Lpc between the H-GOS and CON groups. (**K**,**L**) Changes in the Sl and Cer between the H-GOS and CON groups.

KEGG pathway enrichment analysis demonstrated that the differential metabolites between L-GOS and CON groups were significantly enriched in multiple fatty acid metabolism pathways (Figure S1A). These differential lipids and lipid-like molecules primarily encompass phosphatidylethanolamine (Pe), phosphatidylcholine (Pc), sphingolipids (Sl), and their downstream metabolites, including Lysophosphatidylethanolamine (Lpe), Lysophosphatidylcholine (Lpc), and ceramide (Cer) (Figure 6A). Specifically, compared to the CON group, the abundances of seven Pcs, such as Pc(p-16:0/0:0), Pc(o-18:0/0:0), Pc(20:4/0:0), Pc(p-18:0/0:0), Pc(16:0/20:4), Pc(20:3/0:0), and Pc(22:6/0:0), and six Pes, such as Pe(p-16:0/0:0), Pe(p-18:0/0:0), Pe(o-16:0/0:0), Pe(o-16:2/2:0), Pe(o-16:4/6:0), and Pe(p-16:0/20:5), were significantly down-regulated in L-GOS mice (Figure 6B,C). Simultaneously, abundances of nine Lpcs and fourteen Lpes were also significantly decreased in L-GOS mice (Figure 6D,E). In addition, two Sls, namely Sl(18:0_o/15:0) and Sl(16:0_o/15:0), were significantly up-regulated in L-GOS mice. Meanwhile, abundances of fifteen Cers and their phosphorylated derivatives, such as Cer[ns](d32:1), Cer(16:0_20/16:2), Cer(18:1_20/20:2), Cer[ap](t36:0), Cer(18:1_20/12:0), Cer(19:2_20/14:0), Cer(9:1_20/20:3), CerP(15:1_20/12:0), CerP(18:0_20/2:0), CerP(15:1_20/10:0), CerP(d17:0/2:0), CerP(d19:1/2:0), CerP(15:1_20/14:0), CerP(d18:1/2:0), and CerP(14:1_20/14:0), were significantly down-regulated in L-GOS mice (Figure 6F–H). Although some changes in phospholipid metabolites were detected between the H-GOS and the CON groups, the number of changes was fewer than that in the L-GOS group (Figure 6I,J). Specifically, abundances of three PCs, three LPCs, and five Cers were significantly reduced, while abundances of 3 Sls were significantly increased (Figure 6K,L).

### 3.5. GOS Supplementation Modulates the Cecal Composition of Aromatic Amino Acids in Mice

**Figure 7:**
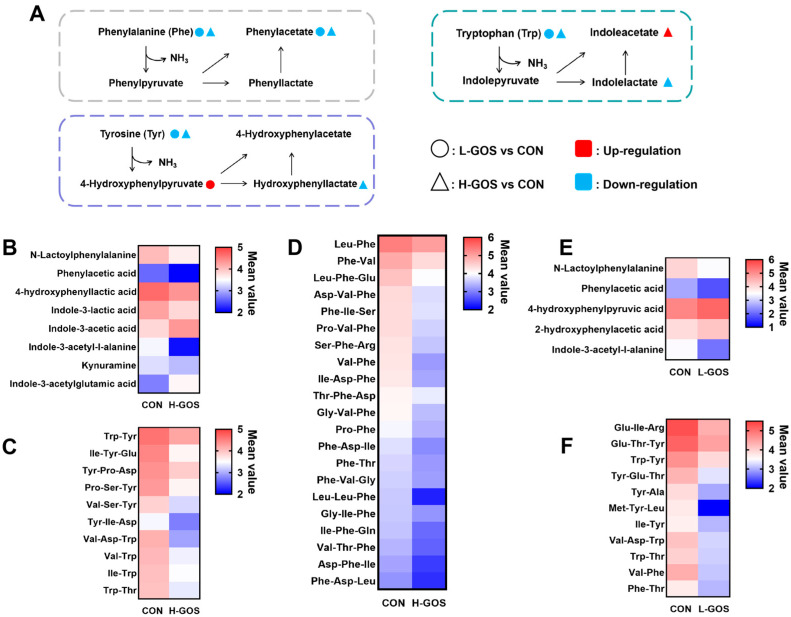
The effects of GOS supplementation on the cecal metabolic derivatives of aromatic amino acids in the mice (*n* = 6). (**A**) Schematic overview of important metabolites and major metabolic pathways associated with aromatic amino acid metabolism. Circle represents L-GOS vs. CON; triangle represents H-GOS vs. CON; red represents up-regulated; blue represents down-regulated. (**B**) Changes in the metabolic derivatives of aromatic amino acids between the H-GOS and CON groups. (**C**,**D**) Changes in the short peptides containing tyrosine, tryptophan and phenylalanine between the H-GOS and CON groups. (**E**) Changes in the metabolic derivatives of aromatic amino acids between the L-GOS and CON groups. (**F**) Changes in the short peptides containing tyrosine, tryptophan and phenylalanine between the L-GOS and CON groups.

KEGG pathway enrichment analysis further demonstrated that signaling pathways including tyrosine metabolism and tryptophan metabolism were significantly enriched in GOS-treated groups (Figure S1B). Notably, seven gut bacterial-derived aromatic amino acid metabolites exhibited altered abundances between H-GOS and CON groups (Figure 7A,B). Compared to the CON group, abundances of N-Lactoylphenylalanine, phenylacetic acid, 4-hydroxyphenyllactic acid, Indole-3-lactic acid, and Indole-3-acetyl-L-alanine were significantly reduced in the H-GOS group, whereas the abundances of Indole-3-acetylglutamic acid and Indole-3-acetic acid were significantly elevated. Additionally, abundances of 31 small peptides containing phenylalanine, tyrosine, or tryptophan were significantly decreased in the H-GOS group (Figure 7C,D). Similarly, abundances of five gut bacterial-derived aromatic amino acid metabolites were altered between the L-GOS and CON groups. As shown in Figure 7E, compared to the CON group, cecal abundances of N-Lactoylphenylalanine, phenylacetic acid, and Indole-3-acetyl-L-alanine were significantly reduced in the L-GOS group, while abundances of 4-hydroxyphenylpyruvic acid and 2-hydroxyphenylacetic acid were significantly increased. Meanwhile, abundances of 11 small peptides containing phenylalanine, tyrosine, or tryptophan were significantly decreased in the L-GOS group (Figure 7F).

## 4. Discussion

To investigate whether GOS supplementation modulates the production of gut microbiota-derived metabolites beyond SCFAs, the present study systematically evaluated the effects of GOS at different doses on the microbial community structure and metabolic profiles in the cecal digesta of mice. Consistent with previous findings [21,22,23], GOS supplementation at both low and high doses significantly enriched SCFA-producing bacteria in the cecum. Metabolomic analyses further revealed that L-GOS primarily reduced the concentrations of microbiota-derived Lpe, Lpc, and Cer in the cecum, whereas H-GOS markedly altered microbial metabolic pathways associated with aromatic amino acids. Collectively, these results demonstrate that GOS supplementation not only enhances the production of bacterial-derived SCFAs but also regulates bacterial metabolic activities related to phospholipids, sphingolipids, and aromatic amino acids.

Increased intestinal bacterial richness is generally considered to reflect a more diverse and stable gut microecosystem, which is closely associated with improved overall host health [24]. Consistent with previous findings [11,22], the present study showed that dietary GOS supplementation via drinking water markedly altered cecal microbial community structure and increased its richness in mice. These results indicate that GOS supplementation supports the maintenance of a stable gut microecosystem. Beyond its effects on microbial richness, GOS supplementation significantly modulated the relative abundance of cecal bacteria at the genus level. Specifically, L-GOS intervention significantly enriched the relative abundances of *[Eubacterium] brachy* group, *Faecalimonas*, and *[Eubacterium] siraeum* group in the mouse cecum. Previous studies have confirmed that these three genera are key butyrate-producing bacteria, playing critical roles in maintaining intestinal barrier function and immune homeostasis [25,26]. Additionally, *Christensenellaceae R-7* group and *Coriobacteriaceae UCG-002*, two genera with well-documented anti-inflammatory potential [27], were also significantly enriched in L-GOS-treated mice. Notably, L-GOS supplementation significantly reduced the relative abundances of the opportunistic pathogen *Escherichia-Shigella* and the protein-degrading bacterium *Anaerovorax*. H-GOS intervention also significantly increased the relative abundance of *Faecalimonas*, which is involved in promoting butyrate metabolism [26]. Furthermore, the relative abundance of *Adlercreutzia*, a probiotic genus known to produce acetate and propionate [26,28], was significantly elevated in the H-GOS group. Similar to the L-GOS group, the relative abundance of *Anaerovorax* was significantly decreased in the H-GOS group. Collectively, these findings indicate that GOS supplementation not only promotes the colonization of SCFA-producing bacteria and potential probiotics in the hindgut but also suppresses the overgrowth of protein-degrading bacteria, thereby facilitating the beneficial remodeling of the gut microecosystem.

With the alterations in bacterial community composition, GOS supplementation also significantly changed the metabolic profile of the cecal digesta. Pc consists of glycerol, fatty acid residues, and a choline head group [29]. As the most abundant component of cell membranes, Pc accounts for 40–50% of total cellular phospholipids and is primarily localized in the outer leaflet of the plasma membrane lipid bilayer [29,30]. Intestinal Pc is derived from dietary sources or de novo biosynthesis in the liver and intestinal epithelial cells, followed by secretion into the intestinal lumen [31]. Our results showed that L-GOS treatment significantly reduced the levels of seven Pc species in the mouse cecum. GOS entering the cecum is fermented by bacteria to produce SCFAs, which serve as energy substrates for intestinal epithelial cells and promote epithelial growth [12]. Additionally, a previous study demonstrated that GOS can modulate the cell cycle of the ileal epithelium in piglets [18]. Combining these findings, the decreased cecal Pc levels in the L-GOS group may be attributed to the altered renewal rate of intestinal epithelial cells induced by GOS. Furthermore, *Bacteroides* species in the gut can metabolize Pc to generate Lpc [32]. Although no significant change in abundance of *Bacteroides* was observed, its relative abundance slightly decreased in GOS-treated mice, leading to a significant reduction in cecal Lpc levels. Pe contains ethanolamine as its nitrogenous head group linked to a phosphate residue [29]. As the second most abundant phospholipid in mammalian cells, Pe constitutes 20–50% of total membrane phospholipids [33]. In mitochondrial membranes, Pe accounts for approximately 40% of all phospholipids and 15–25% in other organelles [34]. The present study also found that L-GOS significantly reduced the levels of six Pe species in the cecum. In a previous study, we observed that GOS supplementation enhanced the mitochondrial antioxidant capacity in the liver of piglets [35], which suggests that L-GOS may mitigate mitochondrial damage in the epithelial cells of the hepatointestinal system, ultimately leading to a reduction in cecal Pe levels. Concomitantly, Lpe, the downstream metabolite of Pe, was also significantly decreased in the cecum of the L-GOS group. Notably, although H-GOS also altered the cecal levels of Pc and Pe, its regulatory effects were less pronounced than those of L-GOS. In addition to Pc and Pe, GOS treatment also modified the cecal levels of Sl. The Sl is primarily localized in the plasma membrane and certain organelles, and in the intestinal mucosa, accounting for less than 10% of the total phospholipids [29]. Intestinal Sl is derived from dietary sources and is metabolized in the intestinal lumen by sphingomyelinases and ceramidases, and occurs in the form of ceramide, Pc, sphingosine, and fatty acids [36]. The present results showed that GOS treatment significantly increased the cecal levels of Sl while reducing the levels of Cer, suggesting that GOS may inhibit the degradation of Sl to Cer. Given that Sl exhibits anticancer activity whereas Cer promotes cell apoptosis [37,38], GOS supplementation may enhance intestinal health by modulating Sl metabolism in the gut.

Most dietary proteins and polypeptides are digested in the small intestine to produce short peptides and free amino acids, which are then transported into the systemic circulation [39]. Undigested proteins pass into the hindgut, where they serve as critical fermentable substrates for the gut microbiota and undergo extensive hydrolysis to generate a variety of amino acid derivatives [13]. Notably, the present study demonstrated that GOS treatment markedly altered the metabolic profiles of aromatic amino acids by the cecal microbiota. Accumulating evidence indicates that gut microbiota are deeply involved in the metabolic transformation of aromatic amino acids, in addition to the host-endogenous metabolic pathways [13,40]. Intestinal bacteria can convert phenylalanine, tyrosine, and tryptophan into phenethylamine, tyramine, and tryptamine via decarboxylation, and also mediate their transamination through aromatic amino acid transaminases to generate phenylpyruvate, 4-hydroxyphenylpyruvate, and indole-3-pyruvate, respectively [13,41]. The pyruvate moiety of these ketoacid intermediates can undergo subsequent oxidation and reduction reactions to form the corresponding acetate derivatives (phenylacetic acid, 4-hydroxyphenylacetic acid, indole-3-acetic acid) or propionate derivatives (phenylpropionic acid, 4-hydroxyphenylpropionic acid, indole-3-propionic acid) [13,40]. The present findings revealed that GOS treatment significantly reduced the cecal levels of phenylacetic acid, suggesting the inhibition of bacterial phenylalanine catabolism. Concomitantly, the relative abundance of phenylalanine-metabolizing bacteria such as Escherichia-Shigella and Bacteroides was decreased in the GOS-treated groups. Thus, the GOS-mediated reduction in cecal phenylacetic acid levels may be associated with the diminished availability of phenylalanine substrates and the modulation of phenylalanine-degrading microbial communities. Additionally, L-GOS significantly elevated the cecal levels of 4-hydroxyphenylpyruvate, while H-GOS decreased the concentration of its end product 4-hydroxyphenyllactic acid, indicating that GOS exerts dose-dependent regulatory effects on bacterial tyrosine metabolism. With regard to tryptophan metabolism, H-GOS significantly increased the cecal levels of microbiota-derived indole-3-acetic acid, while the concentration of kynurenamine, a key metabolite of the host-dominant kynurenine pathway, was reduced, suggesting that H-GOS may promote the metabolic diversion of tryptophan toward the microbial metabolic pathway. Recent studies have established that phenylacetic acid can accelerate endothelial cell senescence [42], while indole-3-acetic acid exhibits potent anti-inflammatory properties in the gut [43]. Collectively, these results indicate that GOS may promote intestinal health by reshaping the metabolic network of aromatic amino acids in the gut microbiota and modulating the balance between detrimental and beneficial metabolites.

Several limitations of the present study should be acknowledged. First, the sample size of mice in each experimental group was relatively small (*n* = 6 for metabolomic analysis), which may reduce the statistical power of the findings and limit the generalizability of the observed regulatory effects of GOS on cecal microbiota composition and lipid metabolic profiles in this mouse model. Further studies with larger sample sizes are therefore warranted to validate and extend the current results. Second, this study only investigated the cecal microbiota and metabolic profiles at a single time point after 4 weeks of GOS intervention; dynamic analysis of the gut microbiota and metabolites during the intervention period would provide a more comprehensive understanding of the regulatory effects of GOS. Third, the present study was conducted in healthy mice, and the regulatory effects of GOS on gut microbiota and metabolite profiles in disease models (e.g., inflammatory bowel disease, intestinal barrier damage) remain to be investigated.

## 5. Conclusions

In summary, the present study demonstrates that dietary GOS supplementation at different doses remodels the cecal bacterial community structure and modulates the associated phospholipid and aromatic amino acid metabolic profiles in mice. L-GOS primarily exerts regulatory effects on phospholipid metabolism in the cecum, while H-GOS mainly alters the microbial metabolic pathways of aromatic amino acids. These findings provide novel mechanistic insights and identify potential molecular targets for future research on the application of GOS in intestinal health interventions. Nevertheless, further studies are required to validate whether the GOS-mediated regulatory effects on key phospholipid and aromatic amino acid metabolites ultimately influence host intestinal physiology and health, for example, through studies using disease models or germ-free mice.

## Data Availability

The original contributions presented in the study are included in the article; further inquiries can be directed to the corresponding author. The data of 16S rRNA sequencing and Metabolomics were submitted to the National Genomics Data Center (https://ngdc.cncb.ac.cn/omix/preview/roqfLTGH, accessed on 23 October 2025) and are available in the OMIX with the data set identifier OMIX013866.

## Supplementary Materials

The following supporting information can be downloaded at https://www.mdpi.com/article/10.3390/microorganisms14030652/s1: Supplementary Table S1: Detailed information on differential metabolites between the L-GOS and CON groups; Supplementary Table S2: Detailed information on differential metabolites between the H-GOS and CON groups; Figure S1A: KEGG terms of differential metabolites between the L-GOS and CON groups; Figure S1B: KEGG terms of differential metabolites between the H-GOS and CON groups.
