# Supplementary material for: Effects of Galacto-Oligosaccharide Supplementation on Cecal Microbiota, Phospholipid and Aromatic Amino Acid Metabolism in Mice
Source: Microorganisms. 2026 Mar 13;14(3):652. doi: 10.3390/microorganisms14030652 (PMC13029313; doi:10.3390/microorganisms14030652)
Supplement: Supplementary file 1 [file microorganisms-14-00652-s001.zip › Supplementary Figure S1.pdf]

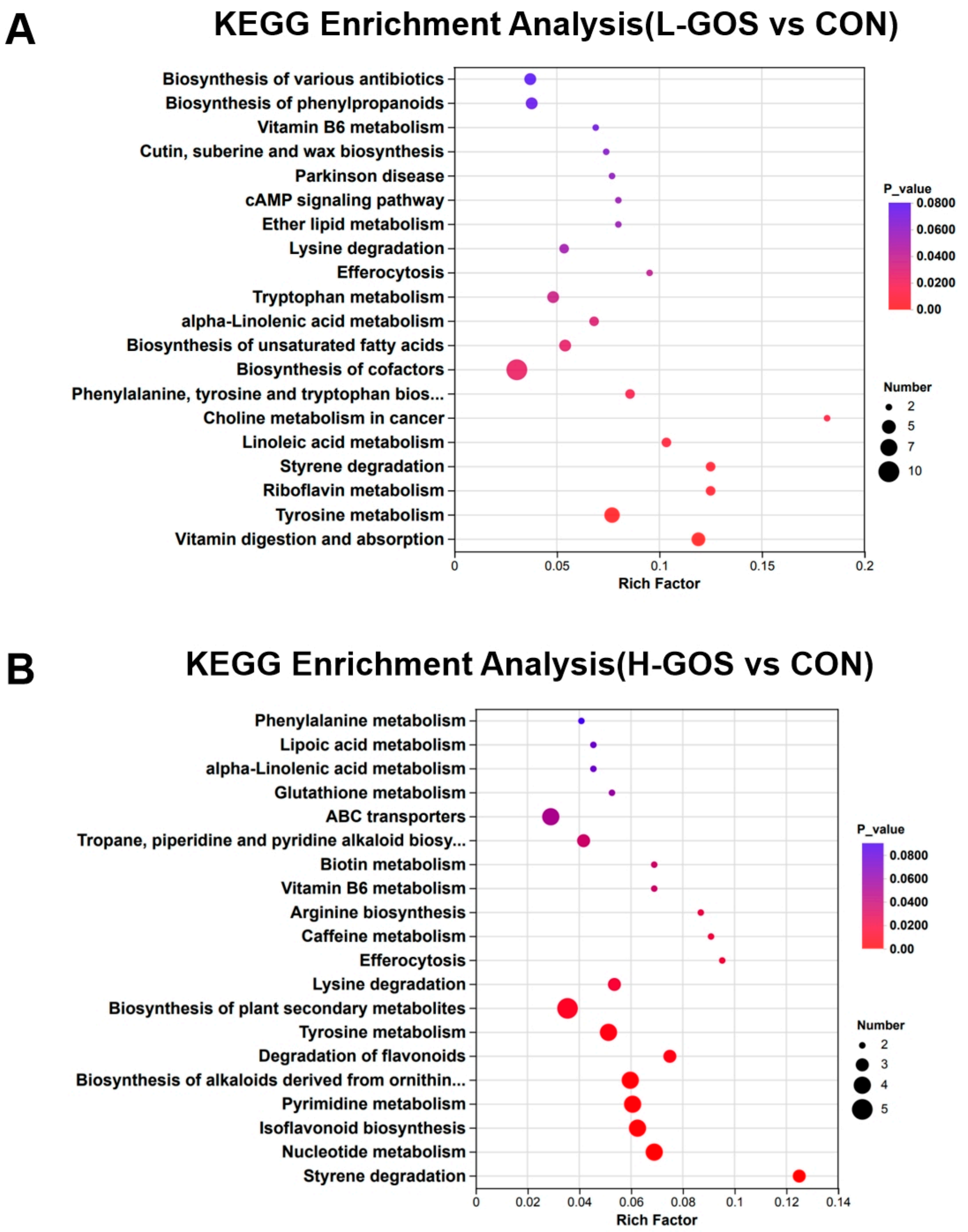

Figure S1. (**A**) KEGG terms of differential metabolites between the L-GOS and CON groups; (**B**) KEGG terms of differential metabolites between the H-GOS and CON groups.
